# Star group´ Mechanical ventilation multicentre snapshot audit and survey

**DOI:** 10.1186/2197-425X-3-S1-A100

**Published:** 2015-10-01

**Authors:** CP Newell, K Oglesby, K Crewdson, M Martin, N Dodds, J Astin, A Grant, A Ray, S Heikal, H Lahie, W Seligman, H Davies, C Bourdeaux, Severn Trainees Anaesthetic Research (STAR) Group

**Affiliations:** Royal United Hospitals Bath NHS Foundation Trust, Bath, United Kingdom; University Hospitals Bristol NHS Foundation Trust, Bristol, United Kingdom; North Bristol NHS Trust, Bristol, United Kingdom; Gloucestershire Hospitals NHS Foundation Trust, Cheltenham, United Kingdom; Gloucestershire Hospitals NHS Foundation Trust, Gloucester, United Kingdom; Weston Area Health NHS Trust, Weston-super-Mare, United Kingdom; Great Western Hospitals NHS Foundation Trust, Swindon, United Kingdom

## Introduction

There is good evidence that all invasively ventilated patients should undergo lung-protective ventilation (LPV) [1, 2]. As well as LPV, there is a strong evidence base for other routine interventions in the care of ventilated patients. We performed a one day snapshot audit and survey of attitudes towards invasive ventilation practice in intensive care units across the Severn (Bristol,UK) region.

## Objectives

1. To determine compliance with the central components of the Institute for Healthcare Improvement (IHI) Ventilator Bundle

2. To audit use of continual waveform capnography in all ventilated patients

3. To audit the use of LPV

4. To concurrently survey the opinions of senior ICU staff towards the management of ventilated patients

## Methods

A 24-hour snapshot audit and survey were conducted in regional Intensive Care Units (ICUs). All invasively ventilated patients were included. Data collected included ventilation parameters, use of waveform capnography, Selective oral decontamination and patient position. The on-call consultant(s) and nurse in charge were surveyed to establish individual opinion regarding the use of invasive ventilation.

Ethical approval was obtained from the local R&D departments prior to starting the study.

## Results

**Figure 1:**
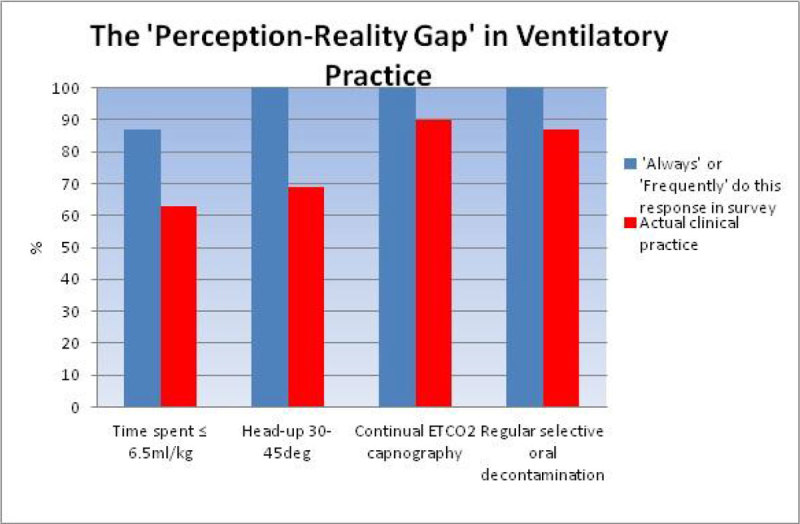
Figure 1

7 of the 8 regional ICUs participated. 39 patients were invasively ventilated during the study period. Of the 18 patients being ventilated in a mandatory mode, 156 data entry points were recorded. 17 (96%) were ventilated with a volume-control mode and 95% had height documented. 63% of the time was spent at ≤6.5 ml.kg^-1^ IBW, the range was 0% to 92% between individual centres. Mean tidal volume was 6.5 ml.kg^-1^ IBW, with significant variability between centres (mean individual centre range 5.6-9.0 ml.kg^-1^ IBW). 23 out of 24 staff completed the survey, 91% felt that all patients should receive LPV and 87% said they either always or frequently ventilated all patients with ≤6 ml.kg^-1^ IBW tidal volumes. Other data is illustrated in Figure 1.

## Conclusions

Considerable variability in ventilation practice exists between the ICU's of this region, particularly in adherence to LPV. There was also a significant disconnect between the care physicians perceived they were delivering and the reality of their practice with low tidal volume ventilation and head-up positioning. The reasons for this are unclear, but have been observed in other similar studies [3]. This questions the validity of surveys alone in assessing clinical practice.
